# Prediction of Life Satisfaction in People with Parkinson's Disease

**DOI:** 10.1155/2020/1561037

**Published:** 2020-07-29

**Authors:** Stina B. Jonasson, Merja Rantakokko, Erika Franzén, Susanne Iwarsson, Maria H. Nilsson

**Affiliations:** ^1^Memory Clinic, Skåne University Hospital, Malmö, Sweden; ^2^School of Health and Social Studies, JAMK University of Applied Sciences, Jyväskylä, Finland; ^3^Department of Neurobiology, Care Sciences and Society, Division of Physiotherapy, Karolinska Institutet, Stockholm, Sweden; ^4^Allied Health Professionals Function, Medical Unit Occupational Therapy & Physiotherapy, Karolinska University Hospital, Stockholm, Sweden; ^5^Department of Health Sciences, Lund University, Lund, Sweden; ^6^Clinical Memory Research Unit, Department of Clinical Sciences Malmö, Lund University, Lund, Sweden

## Abstract

**Introduction:**

People with Parkinson's disease (PD) have lower life satisfaction (LS) than healthy peers. No study has yet identified predictors of LS in people with PD. Such information would be valuable for health care and future interventions that aim to maintain or increase LS.

**Aim:**

To examine how LS evolved in people with PD over a 3-year period, as well as to identify predictive factors of LS.

**Methods:**

We used data from baseline assessments and a 3-year follow-up of 163 people with PD (baseline, mean age 68 years; median PD duration 8 years, 35% women). LS was assessed with item 1 of the Life Satisfaction Questionnaire (LiSat-11). Dichotomized LS data from the 3-year follow-up were used as the dependent variable in multivariable logistic regression analyses. In the first step, independent variables included baseline information on sex, education, general self-efficacy, motor symptoms, perceived walking difficulties, fall-related activity avoidance, and difficulties with/need help in activities of daily living. At the second step, depressive symptoms were added as an independent variable.

**Results:**

The proportion of those who reported being satisfied with their lives reduced from 63.2% at baseline to 49.7% 3 years later (*p*=0.003). When depressive symptoms were not included in the analysis, general self-efficacy (odds ratio, OR = 1.081; 95% CI = 1.019–1.147) and perceived walking difficulties (OR = 0.962; 95% CI = 0.929–0.997) were significant (*p* < 0.05) predictors of LS 3 years later. With depressive symptoms included, the influence of walking difficulties diminished, and depressive symptoms (OR = 0.730; 95% CI = 0.607–0.877) and general self-efficacy (OR = 1.074; 95% CI = 1.010–1.142) were the only significant predictors of LS 3 years later.

**Conclusions:**

LS is reduced over a 3-year period. The study suggests that perceived walking difficulties, general self-efficacy, and depressive symptoms are important predictors of LS in people with PD.

## 1. Introduction

Life satisfaction (LS) has been defined as “the degree to which a person positively evaluates the overall quality of his/her life as a whole” [1]. Aspects that impact LS might differ for various persons depending on their life circumstances. People with Parkinson's disease (PD) have shown significantly lower LS than age- and sex-matched controls [2]. The onset of PD is associated with a marked decline in LS; a previous study reported that PD onset affected LS more negatively than a change in self-rated health from “very good” to “very bad.” Moreover, the effect of PD onset was almost twice as large as the effect of depression on LS [3].

Previous cross-sectional studies have identified factors associated with LS in people with PD [2, 4–6], whereof one [4] used the same cohort as the present longitudinal study. These studies showed that personal factors, such as general self-efficacy [4] and sense of coherence [5], as well as the presence of comorbidities (angina pectoris, cancer, and ulcers/gastritis [2]), were significantly associated with LS. There are conflicting results regarding the impact of PD duration [2, 4, 5], sex [2, 4–6], marital/relationship status [2, 6], and working situation [2, 4–6]. Moreover, there are inconclusive results regarding the association between depressive symptoms and LS in people with PD [4, 5]. However, depressive symptoms affect LS to a great degree in the general population, e.g., [7–9]. Though several studies have shown that personal factors, such as being married or cohabiting [8, 10, 11] and having a good economic situation [7, 12, 13] and higher education [8, 12, 13], are significantly associated with higher LS in the general population, a recent review concluded that the preservation of motor skills is fundamental for maintaining LS in people with PD [14].

To the best of our knowledge, no study has yet identified predictors of LS in people with PD, i.e., by using longitudinal data. Such information would be valuable in health care and for intervention programs aiming at maintaining or increasing LS in people with PD.

The aim of this study was to examine how LS evolved in people with PD over a 3-year period. Moreover, we aimed to identify predictive factors of LS. Building on the knowledge base from previous cross-sectional PD studies, we focused on the impact of personal factors and motor-related aspects. The predictors of LS were identified with and without taking the potential effect of depressive symptoms into account. Based on previous studies in the general population [7–9], our hypothesis was that depressive symptoms is an independent predictor of LS.

## 2. Methods

This longitudinal study is part of the larger project “Home and health in people ageing with PD” (HHPD), which includes baseline assessments (2013) and a 3-year follow-up (2016). Extended information about methods and design of the larger project is available in the study protocol [15].

### 2.1. Participants and Recruitment

Participants were recruited from three hospitals in southern Sweden. Previous publications present flowcharts of the recruitment process for baseline assessments [16] and a 3-year follow-up [17]. A total of 653 persons fulfilled the inclusion criteria of having a PD diagnosis (G20.9) since at least one year. Exclusion criteria were difficulties in understanding or speaking Swedish, severe cognitive difficulties, living outside Skåne County, or other reasons that made them unable to give informed consent or take part in the majority of the data collection (*n* = 216). The remaining 437 persons were invited to participate. Of these, 22 were unreachable, two had a revised diagnosis, and 157 declined. One person was excluded due to extensive missing data, resulting in a sample of 255 participants at baseline.

At the 3-year (±3 months) follow-up, 22 participants were deceased, three had moved, and one ended up outside the follow-up window. Consequently, 229 persons were invited to participate, whereof eight were unreachable, four had a revised diagnosis, and 51 declined. One person was excluded due to extensive missing data and low data quality, resulting in a sample of 165 participants. Of these, one participant did not respond to the question targeting LS, whereas another took more than 60 days to complete the different parts of the data collection. These two persons were excluded from further analyses. Thus, the final sample for the present study comprised 163 participants (35% women). At baseline, their mean (SD; min-max) age was 68.3 (8.8; 45–91) years, and their median (q1–q3) PD duration was 8 (5–12) years. Median (q1–q3) PD severity (assessed during “on-state”) according to Hoehn and Yahr staging (scored 1–5, higher = worse) [18] was 2 (2–3), see Table 1 for further details about the participants' characteristics at baseline.

There was no difference regarding PD duration between the 163 participants and the 92 persons who were lost to follow-up (median 8 years at baseline in both groups; *p*=0.255, Mann–Whitney *U* test). However, the participants were significantly younger and comprised less women than those lost to follow-up: mean 68 vs. 73 years at baseline, *p* > 0.001 (independent samples *t*-test); 35% vs. 49% women, *p*=0.034 (Pearson's chi-square test).

The HHPD project was approved by the Regional Ethical Review Board in Lund, Sweden (nos. 2012/558; 2015/611). All participants gave their written informed consent.

### 2.2. Procedure

Baseline assessments and the 3-year follow-up had a similar procedure and data collection. Both included self-administered questions/questionnaires (administered as a postal survey about 10 days prior to a subsequent home visit), as well as interview-administered questions/questionnaires and clinical assessments carried out during a home visit. The present study used data from the baseline assessments to identify predictors of LS at the 3-year follow-up.

### 2.3. Assessments

#### 2.3.1. Life Satisfaction (Dependent Variable)

LS was assessed with item 1 of the Life Satisfaction Questionnaire (LiSat-11) [19], which was interview-administered during the home visit. Item 1 is posed as follows: “My life as a whole is” and response options are scored from 1–6: very dissatisfying (1); dissatisfying (2); rather dissatisfying (3); rather satisfying (4); satisfying (5); very satisfying (6). For the logistic regression analyses and for longitudinal comparison of the prevalence, LS was dichotomized into 0: not satisfied (scores 1–4) and 1: satisfied (scores 5-6) [4, 19–21].

#### 2.3.2. Personal Factors

Data on personal factors included sex, general self-efficacy, and education level. The General Self-Efficacy Scale (GSE, scored 10–40; higher = better) was included in the self-administered postal survey [22]. Highest education level (university/high school/elementary school, whereof the two latter categories were pooled into one category) was recorded during the interview at the home visit.

#### 2.3.3. Motor-Related Aspects

Data on motor-related aspects included perceived walking difficulties, fall-related activity avoidance, difficulties and dependence in activities of daily living, and motor symptoms. All but motor symptoms were assessed through the self-administered postal survey. The Generic Walk-12 (Walk-12G, scored 0–42; higher = worse) was used to assess perceived walking difficulties in daily life [23]. The Parkinson's disease Activities of Daily Living Scale (PADLS, scored 1–5; higher = worse) was used to assess difficulties and dependence in activities of daily living [24]. PADLS was dichotomized: those who scored ≥3 were classified as having difficulties with, or needing help from others in daily activities [25]. A dichotomous (yes/no) question targeted fall-related activity avoidance: “Do you avoid activities due to the risk of falling?” PD specific motor symptoms were clinically assessed with the motor part of the Unified Parkinson's Disease Rating Scale (UPDRS III, scored 0–108; higher = worse) [26].

#### 2.3.4. Additional Data Collection

The Geriatric Depression Scale (GDS-15, scored 0–15; higher = worse) was administered as an interview during the home visit to assess depressive symptoms [27]. Moreover, data on PD duration were collected during the interview at the home visit (for descriptive purposes).

### 2.4. Statistical Analyses

Participant characteristics at baseline were analyzed using descriptive statistics (means and SD, medians and q1–q3, or percentages, depending on data level), see Table 1. Changes in LS from baseline to the 3-year follow-up were analyzed by using McNemar's exact test for dichotomized data (i.e., scores 1–4 vs. 5-6) and the Wilcoxon signed ranks test for ordinal data.

The relationships between potential independent variables (i.e., baseline values) were investigated by using Spearman's correlation coefficient (*r*_*s*_). The following eight potential independent variables were included: sex, education level, general self-efficacy, motor symptoms, perceived walking difficulties, fall-related activity avoidance, difficulties with/need help in activities of daily living, and depressive symptoms. No signs of multicollinearity were detected, i.e., no correlation was >0.7.

Univariable (simple) logistic regression analyses were used to test the associations between dichotomized LS at the 3-year follow-up (i.e., the dependent variable) and the independent variables. Independent variables with associations to LS with *p* < 0.3 were included in the forthcoming multivariable regression analyses, i.e., in order to avoid leaving out potentially significant variables. All variables fulfilled this criterion (see Table 1).

In the first step of the multivariable analyses, all independent variables that reflected personal factors and motor-related aspects (i.e., not including depressive symptoms) were entered simultaneously (method: enter). The independent variable with the highest nonsignificant *p* value was manually removed. This was repeated until all independent variables in the model had *p* values <0.1 (Model 1). In the second step, the multivariate analysis was reran from the beginning, with depressive symptoms added as an independent variable (Model 2). Both analyses (Models 1 and 2) were conducted with and without controlling for baseline LS. The models that are not controlled for baseline LS identify predictors of LS at the 3-year follow-up. The models that are controlled for baseline LS identify predictors of changes in LS over a 3-year period, i.e., given the LS at baseline.

Odds ratio (OR) and Wald are presented for all independent variables in the final models as indicators of the strength of their effect on LS. For the final models, Nagelkerke's *R*^2^ is presented as a measure of the models' predictive capacity. Hosmer–Lemeshow tests were conducted as an indication of the goodness of fit for the logistic models; a nonsignificant test indicates that the model is well fitted.

The level of statistical significance was set to *p* < 0.05. Exact, two-tailed *p* values are reported. All statistical analyses were carried out in IBM SPSS Statistics, version 26.

## 3. Results

The proportion of those who were satisfied with their life as a whole reduced from 103 out of 163 participants (63.2%) at baseline to 81 out of 163 (49.7%) at the 3-year follow-up (*p*=0.003). In terms of the median (q1–q3) score, LS decreased from 5 (4-5) at baseline to 4 (4-5) at the 3-year follow-up (*p*=0.002).

### 3.1. Associations between Life Satisfaction and Independent Variables

In univariable regression analyses, four out of the eight independent variables were significantly (*p* ≤ 0.003) associated with LS at the 3-year follow-up, i.e., general self-efficacy, perceived walking difficulties, fall-related activity avoidance, and depressive symptoms, see Table 1. All independent variables fulfilled the criterion of *p* < 0.3.

### 3.2. Predictors of Life Satisfaction

#### 3.2.1. Model 1 (Personal Factors and Motor-Related Aspects)

The multivariable analysis that did not take the potential effect of depressive symptoms into account (Model 1) revealed two independent variables that predicted LS 3 years later: general self-efficacy increased (OR = 1.081) the likelihood of good LS three years later and perceived walking difficulties in daily life decreased (OR = 0.962) this likelihood, see Table 2. The model accounted for 15.7% of the variance in LS.

Perceived walking difficulties (OR = 0.961) was the only significant predictor of LS when controlling for baseline LS. This model accounted for 22.1% of the variance in LS.

#### 3.2.2. Model 2 (Personal Factors, Motor-Related Aspects, and Depressive Symptoms)

When also including depressive symptoms in the multivariable model, the model revealed two independent variables that predicted LS 3 years later: depressive symptoms reduced (OR = 0.730) the likelihood of good LS three years later, whereas general self-efficacy increased (OR = 1.074) this likelihood (Table 3). The model accounted for 24.0% of the variance in LS.

Depressive symptoms (OR = 0.758) was the only significant predictor of LS when controlling for baseline LS. This model accounted for 27.4% of the variance in LS, but a significant (*p*=0.027) Hosmer and Lemeshow test indicated that the data did not perfectly fit the logistic model.

## 4. Discussion

To the best of our knowledge, this is the first study reporting longitudinal data on LS in people with PD, showing that LS is significantly reduced over a 3-year period. The results reveal that both personal factors (i.e., general self-efficacy) and motor-related aspects (i.e., perceived walking difficulties) can predict LS three years later. As hypothesized, depressive symptoms seem to affect LS in people with PD.

The study identifies general self-efficacy as a predictive factor of LS in people with PD, which corroborates prior cross-sectional findings in the same cohort [4]. General self-efficacy refers to a person's belief in their capacity to handle various situations [28], a belief in their ability to respond to and control environmental demands and challenges [22]. Those with low self-efficacy are more likely to give up when facing difficulties, whereas those with high self-efficacy put in greater effort to master challenges [28]. Therefore, people with high general self-efficacy might lead more active lives (physically and socially) than those with low self-efficacy. Indeed, being active has been reported as crucial for LS among people with PD [29], and low self-efficacy is a barrier for being physically active and exercise among people with PD [30, 31]. Self-efficacy is affected by support from family, friends, and the health care system [32], and high self-efficacy is positively correlated with self-management [33]. Thus, self-management programs might be useful to increase self-efficacy and thereby potentially enhance or maintain LS.

Perceived walking difficulties are shown to independently predict LS. However, when also including depressive symptoms, walking difficulties are no longer significant. This corroborates our previous cross-sectional findings [4]. This might indicate that depressive symptoms partly explain the association between perceived walking difficulties and LS. Interestingly, depressive symptoms do not predict perceived walking difficulties in people with PD (submitted). The potential importance of perceived walking difficulties in relation to LS should be acknowledged in PD care. Future studies are needed to explore whether interventions that target perceived walking difficulties have positive effects on LS.

This study identifies depressive symptoms as a predictor of LS. The importance of depressive symptoms in relation to LS is in line with a cross-sectional study in the same cohort [4], as well as a longitudinal study of community-living elderly people without PD [9]. However, the current finding is in contrast with the cross-sectional PD study by Rosengren et al. [5], based on a similar study sample (mean age 70.1 vs. 68.3 years; PD duration mean 7.4 vs. median 8 years). The discrepancy in findings might be explained by the fact that they used dichotomized data of an adapted version of the GDS-15 with 5 additional items, covering symptoms of insomnia, anxiety, panic, pain, and hypochondria [34]. These can be symptoms of other disorders and might not be related to depressive symptoms per se. Moreover, the expanded version was developed for older patients in primary care centers [34] and has to our knowledge not been evaluated for use in PD. The discrepancy might also reflect that LS was not assessed in the same way as in the present study [5]. Regardless, depressive symptoms are common in people with PD, but recognition might be hindered by somatic PD symptoms [35]. Recognition and treatment of depressive symptoms might be one way to maintain LS in people with PD.

At the 3-year follow-up, the level of LS was identical to the LS score in a previous Swedish PD study by Gustafsson et al. [2], whereas their median PD duration was 6 years, compared to 11 years in the present study. These findings suggest that although PD onset greatly affects LS [3], disease duration has no strong impact on LS in people with PD. Our finding that LS is significantly reduced over time might instead be a result of worsened PD symptoms. PD is a vastly heterogeneous disease [36], and although Gustafsson et al. had a study sample with lower PD duration than in the present study, this is no guarantee for milder PD. Unfortunately, Gustafsson et al. did not report any measures of PD severity [2].

### 4.1. Strengths and Limitations

Strengths of this study include the longitudinal design, which allowed the identification of predictive factors. Furthermore, the use of multivariable regression analyses is a strength because relying solely on univariable analyses does not always provide reliable results, as the results are often changed when the complexity of several variables is considered simultaneously (as in real life). However, due to a limited sample size, we were only able to study the predictive value of eight independent variables. The Nagelkerke *R* square for the multivariable regression analyses varied from 0.157 to 0.274, implying that the models account for 15.7–27.4% of the variation in LS at the 3-year follow-up. That is, there are factors not addressed in the present study that also affect LS. Future studies are required to explore additional variables that might influence LS in people with PD, such as PD severity, fatigue, and social relations.

We used dichotomized data, both as the dependent variable and for some of the independent variables in our analyses. Dichotomization of data implies that some information is lost. However, due to uneven scoring distribution across the scale, it was not possible to use LS as an ordinal variable. This was the case also for some of the independent variables. Moreover, if we had not dichotomized some of the independent variables, we would had been even more limited regarding the number of independent variables that could be included in the analyses, i.e., as using variables with more than two categories requires a larger sample size.

It should be noted that the participants (i.e., those who completed assessments at both baseline and the 3-year follow-up) were somewhat younger than those lost to follow-up. However, there was no significant difference in PD duration. There are dropouts in all longitudinal studies, which might hamper the external validity of the findings.

The Hosmer and Lemeshow test was significant for the regression model that included depressive symptoms, when controlling for LS at baseline. This implies that the data do not perfectly fit the logistic model. However, we performed additional analyses of the GDS-15, which confirmed a linear relationship between the odds ratio of GDS-15 and LS at the 3-year follow-up. The model and these additional analyses were discussed with a statistician (PhD), who concluded that the model fit was satisfactory. Nonetheless, odds ratios for this model should be interpreted with caution.

## 5. Conclusions

In this first longitudinal study of LS in people with PD, LS is reduced over a 3-year period. The study suggests that perceived walking difficulties, general self-efficacy, and depressive symptoms are important predictors of LS. Future studies are needed to replicate these findings, and it would be of interest to investigate whether interventions targeting these factors can maintain or improve LS in people with PD.

## Figures and Tables

**Table 1:** Participants' characteristics at baseline for the total sample and separate for those not satisfied vs. satisfied with their lives at the 3-year follow-up and univariable logistic regression analyses with life satisfaction (3-year follow-up) as the dependent variable, *N* = 163^1^.

Independent variables	Descriptives		Univariable regression analyses (total sample)	
	Total sample	Not satisfied/satisfied	OR (95% CI)	*p* value
Sex (male), *n* (%)	106 (65.0)	49 (59.8)/57 (70.4)	0.625 (0.326–1.198)	0.157
Education (university level), *n* (%)	61 (37.4)	35 (42.7)/26 (32.1)	1.575 (0.831–2.987)	0.164
General self-efficacy (GSE), mean (SD)	30 (6.4)	28 (6.5)/32 (5.7)	1.114 (1.053–1.178)	**<0.001**
Motor symptoms (UPDRS III), mean (SD)	30 (13.5)	31 (12.5)/28 (14.4)	0.986 (0.962–1.010)	0.226
Walking difficulties (Walk-12G), mean (SD)	15 (10.7)	18 (10.8)/12 (9.8)	0.947 (0.917–0.979	**0.001**
Fall-related activity avoidance (yes), *n* (%)	61 (37.4)	40 (48.8)/21 (25.9)	0.368 (0.190–0.711)	**0.003**
Difficulties with, or need help in daily activities (PADLS; yes), *n* (%)	38 (23.3)	23 (28.0)/15 (18.5)	0.583 (0.278–1.221)	0.153
Depressive symptoms (GDS-15), median (q1–q3)	2 (1–4)	3 (2–5)/1 (0–2)	0.707 (0.597–0.838)	**<0.001**

OR = odds ratio; CI = confidence interval; GSE = General Self-Efficacy Scale (10–40; higher = better); UPDRS III = Unified Parkinson's Disease Rating Scale, motor part (0–108; higher = worse); Walk-12G = Generic Walk-12 (0–42; higher = worse); PADLS = Parkinson's Disease Activities of Daily Living Scale (1–5; higher = worse; those who scored >2 were classified as having difficulties or needing help); GDS-15 = Geriatric Depression Scale (0–15; higher = worse). ^1^Except for GSE, UPDRS III, Walk-12G, and GDS-15, which had 1–5 missing cases each. Life satisfaction was assessed with item 1 of the Life Satisfaction Questionnaire (scored 1–6; higher = better), and scores were dichotomized into not satisfied (1–4; coded as 0) and satisfied (5-6; coded as 1). Statistically significant *p* values (0.05) are presented in bold.

**Table 2:** Model 1: multivariable logistic regression analyses with life satisfaction (3-year follow-up) as the dependent variable (personal factor and motor-related aspects as independent variables).

Independent variables	Unadjusted for life satisfaction at baseline, *n* = 158			Adjusted for life satisfaction at baseline, *n* = 159		
	OR (95 % CI)	Wald	*p* value	OR (95 % CI)	Wald	*p* value
General self-efficacy (GSE)	1.081 (1.019–1.147)	6.765	**0.009**			
Walking difficulties (Walk-12G)	0.962 (0.929–0.997)	4.725	**0.030**	0.961 (0.929–0.994)	5.401	**0.020**
Nagelkerke *R* square			0.157			0.221
Hosmer and Lemeshow test, *p* value			0.203			0.534

OR = odds ratio; CI = confidence interval; GSE = General Self-Efficacy Scale (10–40, higher = better); Walk-12G = Generic Walk-12 (0–42, higher = worse). Life satisfaction was assessed with item 1 of the Life Satisfaction Questionnaire (scored 1–6; higher = better), and scores were dichotomized into not satisfied (1–4; coded as 0) and satisfied (5-6; coded as 1). The following 7 independent variables were initially included in the models (backward method): sex; education; general self-efficacy; motor symptoms; walking difficulties; fall-related activity avoidance; difficulties with, or need help in activities of daily living. Statistically significant *p* values (0.05) are presented in bold.

**Table 3:** Model 2: multivariable logistic regression analyses with life satisfaction (3-year follow-up) as the dependent variable (personal factor, motor-related aspects, and depressive symptoms as independent variables).

Independent variables	Unadjusted for life satisfaction at baseline, *n* = 157			Adjusted for life satisfaction at baseline, *n* = 158		
	OR (95 % CI)	Wald	*p* value	OR (95 % CI)	Wald	*p* value
Depressive symptoms (GDS-15)	0.730 (0.607–0.877)	11.310	**0.001**	0.758 (0.633–0.908)	9.123	**0.003**
General self-efficacy (GSE)	1.074 (1.010–1.142)	5.224	**0.022**			
Nagelkerke *R* square			0.240			0.274
Hosmer and Lemeshow test, *p* value			0.660			**0.027**

OR = odds ratio; CI = confidence interval; GDS-15 = Geriatric Depression Scale (0–15, higher = worse); GSE = General Self-Efficacy Scale (10–40, higher = better). Life satisfaction was assessed with item 1 of the Life Satisfaction Questionnaire (scored 1–6; higher = better), and scores were dichotomized into not satisfied (1–4; coded as 0) and satisfied (5-6; coded as 1). The following 8 independent variables were initially included in the models (backward method): sex; education; general self-efficacy; motor symptoms; walking difficulties; fall-related activity avoidance; difficulties with, or need help in activities of daily living; depressive symptoms. Statistically significant *p* values (0.05) are presented in bold.

## Data Availability

All data are archived according to the Swedish Act concerning the Ethical Review of Research Involving Humans to attain confidentiality. Data can be shared with a qualified researcher upon reasonable request, following an approval by the responsible ethical committee.
